# The Plague in Crete During the 19th Century

**DOI:** 10.7759/cureus.42284

**Published:** 2023-07-21

**Authors:** Ioannis Stefanogiannis, Spyros N Michaleas, Eleni Papadaki, Ioannis Mouzas, Marianna Karamanou

**Affiliations:** 1 Department of Medicine, University of Crete Medical School, Heraklion, GRC; 2 Department of History of Medicine and Medical Ethics, National and Kapodistrian University of Athens School of Medicine, Athens, GRC

**Keywords:** history of medicine, yersinia pestis, bubonic, black death, fleas

## Abstract

The plague is one of the most dangerous infectious diseases that can affect mankind. The disease has caused countless pandemics over the centuries in many parts of the world, mainly Asia, Africa, and Europe, and has caused over 200 million deaths, making it one of the greatest scourges of mankind throughout the ages. Similar to the rest of Greece, Crete was affected for many years by the plague during the 19th century, which caused significant mortality, both in the cities and the countryside. The lack of doctors, the absence of organized health systems, the ignorance of the origin and modes of transmission, and the belief of the island’s Muslim conquerors in destiny and God-given diseases made the spread of the plague very easy, while simultaneously making its control, with measures to protect public health, extremely difficult. This led to the repeated decimation of the island's population, with immeasurable social and economic consequences for its progression and future development.

## Introduction and background

The plague is an acute infectious disease caused by the bacterium *Yersinia pestis*, which affects people bitten by fleas (mainly by the species *Xenopsylla cheopis*) that parasitize various rodents, draining their infected blood [1]. The bacterium that causes the plague reproduces extremely rapidly in rodents, which are major reservoirs of the disease and infect human populations by direct or indirect contact. The bacterium enters the human body and circulates through the bloodstream to various organs, where it causes hemorrhagic or necrotic lesions. Human-to-human transmission takes place mainly through respiratory droplets [2].

The plague is a severe pathological condition that occurs in three forms: bubonic, pneumonic, and septicemic. Bubonic plague is the most common form and is characterized by the presence of buboes, usually located in the armpit, groin, or neck. Pneumonic plague is the most dangerous and contagious form, as it affects the lungs. Septicemic plague occurs when the bacteria multiply in the bloodstream, leading to severe sepsis. Plague is characterized by the sudden onset of fever, shivering, headaches, malaise, diarrhea, vomiting, lymph node enlargement, skin lesions, neurological manifestations, and severe general health conditions [3,4]. Despite the availability of a variety of treatment protocols, the plague’s death rate remained high, especially in isolated areas of underdeveloped countries, where there is inability to be diagnosed and treated due to inadequate laboratory and clinical infrastructure. The plague has been known since the ancient times and was probably the plague of Athens, as described by the historian Thucydides (460-400 BC) [5].

## Review

The spread of the disease in Crete

Infectious diseases have been the main cause of death for many populations over the centuries. The lack of scientific knowledge, the rampant spread of the diseases, and the lack of both prevention and treatment have often resulted in fatal outcomes and the decimation of the population of entire regions. It is reported that the infamous "Black Death," caused by the bacterium *Yersinia pestis*, wiped out up to 60% of the European population between 1346 and 1353 AD [6].

**Figure 1:**
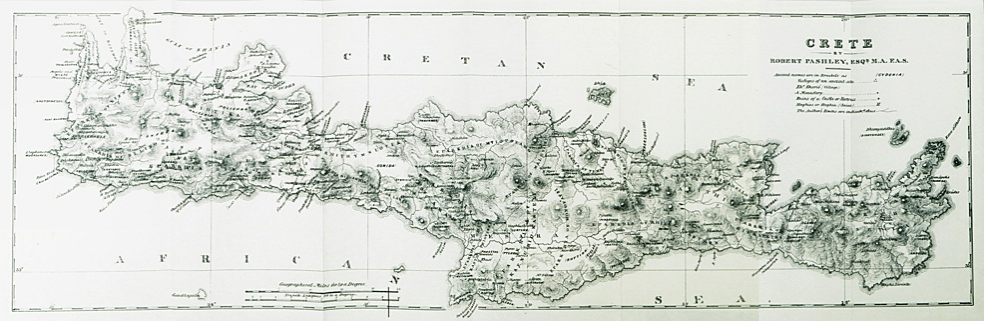
Map of Crete by Robert Pashley, 1837 Source: Private collection.

The first reports of the emergence of plague in Crete date back to 1,400 BC, which mention that the disease was transmitted by the Philistines, whereas the historian Herodotus (484-425 BC) mentioned cases after the period of the Trojan War. The first case of a plague epidemic in Crete was reported by the Venetian historian Vincenzo Maria Coronelli (1650-1718) and concerns the year 251 AD, while Procopius (500-565) also described a great epidemic throughout the entire Byzantine State in 542 AD, during the reign of Emperor Justinian (482-565). Epidemics of plague in Crete were also described in 1330 by the theologist Nicolaos Papadopoulos-Comnenos (1655-1740) in Handakas (Chandax, Heraklion), using Venetian sources in the city of Rethymnon in 1340 and in Handakas in 1376, by Hippolyte Noiret (1864-1888) in 1389 throughout the entire island, and in a document of the Most Serene Republic of Venice in 1398, which caused the extermination of the garrison of Handakas. In addition, plague epidemics in Crete were described by Theocharis Detorakis (1936-2023) in the year 1408, a plague which was transmitted by a ship from Karpathos and caused 15,000 deaths, by Leimonos monastery of Lesvos codex in 1416, by an ecclesiastical codex of Patmos in 1456, by Hippolyte Noiret (1864-1888) in 1465, by a codex from Paris in 1522, by a folk poem in 1571, by the monastery of Apezanon in 1592, by Aristotelis Kouzis (1872-1961) in 1611, by the General Commander of Crete Kontarini in 1630, by a Patmos codex in 1646, from a historical Ottoman document in the year 1655, by the monastery of Dionysios Charalambous on Mount Athos in the year 1678, and by the bishop of Avlopotamos Gedeon (1713-1753) in the year 1718. Other documents, mainly in the Turkish Archives of Heraklion, describe epidemics on the island during the years 1739, 1759, 1770, 1793, 1796, 1810, and 1817 (Figure 1) [7].

However, the data on infectious diseases on the island in the 19th century are incomplete and fragmentary. The most widespread source of information for many years had been the reports and accounts of European travelers visiting the "exotic" Greece of the Ottoman Empire. In the medical books of this period, however, which were published mainly in Vienna and Venice, there are references to the spread of the plague as a very important public health issue [8].

Up until approximately the middle of the 19th century, the plague had been a scourge for the populations of Crete’s towns and villages and kept occurring on a periodic basis [9]. One of the first plague pandemics broke out in Handakas in 1810, right after the hit of an earthquake on the 5th of February. As the author Stefanos Xanthoudidis (1864-1928) informed us, "On February 5, 1810, a terrible earthquake occurred, destroying two-thirds of the city and killing about three thousand people, as it is said. At the same time, the inhabitants of Handakas were struck by a terrible plague, which devastated the town to such an extent that the main streets were completely abandoned...." It was reported that the disease was in fact brought to Crete by Egyptian troops, which had been sent by the viceroy Mehmet Ali (1749-1849) to help the Turks deal with the revolution on the island [10]. 

Furthermore, the French traveler Victor Raulin (1819-1905) reported that, from 1821 until 1839, the plague appeared almost every year. He considered that the Egyptian fleet docked in the port of Souda the main source of transmission, as well as the soldiers' quarters, where there was great overcrowding and unsanitary living conditions [11]. Generally, the main outbreaks of the disease in the 19th century took place in Istanbul and Egypt, both of which maintained strong links with Crete through maritime communications, thus explaining the easy and repeated spread of the disease on the island [12].

**Figure 2:**
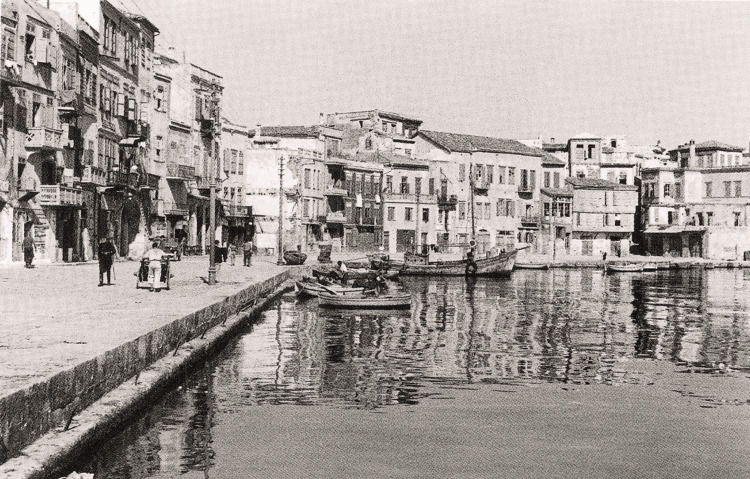
The city of Chania, Crete Source: Private collection.

The Austrian doctor-botanist and traveler Francis Sieber (1789-1844), who visited and stayed on the island throughout 1817, reported a plague epidemic in the area of Kissamos in the prefecture of Chania, where population losses exceeded 50%. In his book, he also mentioned that the bishop of Chania showed him the scars left by the disease, which he managed to overcome, but did not happen to several other people among his relatives and associates (Figure 2) [13].

The plague in the revolution of 1821

During the revolution of 1821, in which Greece was freed from the Ottoman Empire, after almost four centuries, due to the poor sanitary conditions, living in the countryside and in caves, poverty, lack of medical scientists, and absence of an organized health system, dangerous and potentially fatal epidemics of diseases, such as plague, malaria, typhus, dysentery, cholera, and smallpox, often occurred. These diseases are thought to have caused more deaths than the battlefield casualties.

Around the beginning of the revolution of 1821, the plague was widespread in Chania and even more so in the area of Megalo Kastro. Teacher and politician Zacharias Praktikidis (1784-1845) reported that, in order to combat the plague, even the Holy Belt of the Virgin Mary and other holy relics were brought to Crete by monks from the Vatopedi Monastery on Mount Athos. The epidemic was also spreading widely throughout the entire island of Crete [14]. Writer and historian Kallinikos Kritovulidis (1792-1868) reported that of the 1,800 Turks who were imprisoned in the fortress of Kissamos, only 600 surrendered to the Cretan rebels, while the rest were decimated by the plague [15].

In the siege, also, of the fortress of Kantanos, in the south of Chania, it is said that the Turks were forced to capitulate and evacuate the village, mainly because they had a large number of patients and deaths caused by the plague epidemic. It should be noted that the well-known doctor of the time, Stefanos Kanellos (1792-1823), who was committed to the fight against the disease, finally succumbed to it [16]. French traveler Victor Raulin (1819-1905) also mentioned that, from 1821 to 1839, the plague periodically appeared in Crete, with the usual source of transmission being the port of Souda, where the Egyptian and Turkish fleet sailed [17].

Prevention and treatment

**Figure 3:**
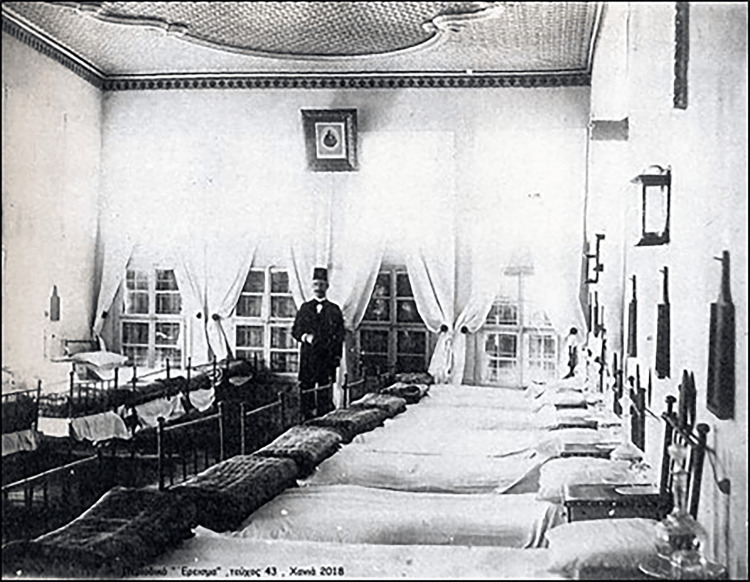
The Chania Hospital in 1885 on Halidon Street Source: Collection of Manolis Manousaka, c. Ereisma (2018, vol. 43).

In general, preventive measures, such as quarantine, were rudimentary or even non-existent by the Turkish conquerors of Crete, who fatalistically believed in untouchable God-given diseases. However, the situation changed during the period of Egyptian rule (1830-1840), when sanitary hospitals were established in the ports of Crete [18]. By way of illustration, French traveler of this period, Guillaume-Antoine Olivier (1756-1714) stated that “Muslims, believing that man cannot alter God’s will, consider any precaution against horrible disease a crime. And while the epidemic mows them down by the thousands, they maintain their serenity and accept death with equanimity. No Turk hesitates to treat his sick relatives with the plague....” This situation, however, does not only affect the Muslim population but also the local population, since Crete was under Turkish occupation throughout the 19th century, with the exception of the decade of the Egyptian occupation. It is also reported that Christian populations often resorted to religion to stop the spread of the plague, with prayers, exorcisms, and processions of images of saints. The disease continued to be endemic in Crete from 1817 to 1839, causing many deaths, including those of Bishops Gedeon and Kallinikos (Figure 3) [19]. 

Treatment options were very limited in the 19th-century Crete. For many years, it was considered that epidemics, such as the plague, were caused by polluted air from the breath of the sick and foul fumes, and so various empirical measures were proposed, such as burying the dead very deep in the earth and covering them with lime, phlebotomy for the removal of the ''bad'' blood, and burning aromatic wood in various parts of the cities. In other instances, a reference was made to the Hippocratic position, which held that epidemics were due partly to internal causes and more to prolonged dampness infecting the veins, while at other times, the exhaustion of the combatants during the war was claimed to facilitate the spread of the disease. In any case, however, it was observed that the removal and isolation of the healthy population was the only way to protect them against the transmission of the plague, and no source mentions partial or complete cure of the disease [20].

More generally, efforts to implement preventive measures were difficult, due to the lack of education and health systems, constant revolutions, the administration’s failure to communicate with the local population, the indifference and inadequacy of government employees, the fatalism, and the general depreciation of the human personality and life.

## Conclusions

The population of Crete in the 19th century was completely defenseless against the plague, which caused thousands of deaths, both in the big cities and in the countryside, because not only the low level of the public health but also of the entire health system, as well as the dramatic living conditions of the population, rendered the actions that aimed to limit the disease ineffective or even unfeasible.

The plague disease in the Turkish-occupied island was accompanied by a significant decimation of local populations. Under the prevailing social, religious, and health conditions, both the prevention and treatment of the disease were extremely difficult, resulting in its frequent and rapid spread throughout the island. However, health conditions improved some decades later, when the disease finally began to be treated in scientific terms and was eventually eradicated.
